# Phenolic constituents from *Alisma plantago*-*aquatica* Linnaeus and their anti-chronic prostatitis activity

**DOI:** 10.1186/s13065-017-0350-9

**Published:** 2017-11-21

**Authors:** Ya-sheng Huang, Qi-qi Yu, Yin Chen, Min-jie Cheng, Li-ping Xie

**Affiliations:** 1 Department of Urology, First Affiliated Hospital, School of Medicine, Zhejiang University, 79 Qingchun Road, Hangzhou, 310003 China; 2 Department of Urology, Hangzhou Hospital of Traditional Chinese Medicine, Hangzhou, 310006 China

**Keywords:** *A. plantago*-*aquatica*, Plantain A, Chronic prostatitis

## Abstract

**Background:**

The plant *Alisma plantago*-*aquatica* Linnaeus, which is widely distributed in southwest of China, is the main material of traditional Chinese medicine “Zexie”. It was used as folk medicine for immune-modulation, anti-tumor, anti-inflammatory and antibacterial. Previous chemical studies on *A. plantago*-*aquatica* reported the identification of triterpenes, diterpenes, sesquiterpenes, steroids, alkaloids and phenolic acid. Terpenes and phenolic acid were regard as major secondary metabolites from this medicine plant.

**Results:**

A new phenolic acid, plantain A (**1**), along with four known compounds (**2**–**5**) were isolated and identified from *A. plantago*-*aquatica* by extensive chromatographic and spectrometric methods. In the present study, the levels of TNF-α, IL-1β, COX-2, PEG2 and TGF-β1 were increased in model group rats, whereas on treatment with the isolated compound (**1** and **4**) at 50 mg/kg, there was a significant decrease in the cytokine levels. Therefore, the anti-CNP effect of **1** and **4** may be related to their anti-inflammatory properties.

**Conclusions:**

A new phenolic acid and four known phenolic compounds were isolated from *A. plantago*-*aquatica*. Moreover, compounds **1** and **4** shows significant anti-chronic prostatitis activity in rats.

**Electronic supplementary material:**

The online version of this article (10.1186/s13065-017-0350-9) contains supplementary material, which is available to authorized users.

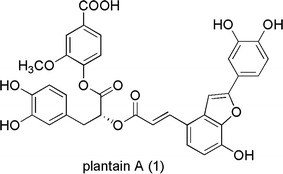

## Background

Prostatitis is a common urological disease causing urination abnormalities, including urinary urgency, frequent urination, micturition, and dysuria. It also can cause suprapubic, lumbosacral, and perineum pain, together with sexual dysfunction, which is also known as prostatitis syndrome. Prostatitis is responsible for up to 2 million outpatient clinic visits per year, including 8% of all male visits to an urologist and 1% of men presenting to primary care physicians [1–3]. Cernilton is one of the most widely used drugs for treating chronic non-bacterial prostatitis, but has not achieved significant curative effect in clinic. Recently, more herbal medicine has being used as alternative therapy for prostatitis [1, 2, 4–6]. Due to its natural constituent and availability, natural herbs which obtained from natural sources are believed to provide less untoward effect profiles and provide greater effectiveness as compared to synthetic drug available over the market.

The plant *A. plantago*-*aquatica*, which is widely distributed in southwest of China, is the main material of traditional Chinese medicine “Zexie”. It was used as folk medicine for immune-modulation, anti-tumor and antibacterial [7–9]. Previous studies on this plant revealed that the water extract of *A. plantago*-*aquatica* showed significant anti-chronic prostatitis activity in rats [2]. To further investigate the constituents and screen the bioactive constituents from this herbal medicine, a phytochemical study was performed that resulted in the isolation of one new compound, along with four known phenolic components. Herein, we report the isolation, structural elucidation, and anti-chronic prostatitis activity of compounds **1**–**5**.

## Results and discussion

### Chemistry

**Fig. 1:**
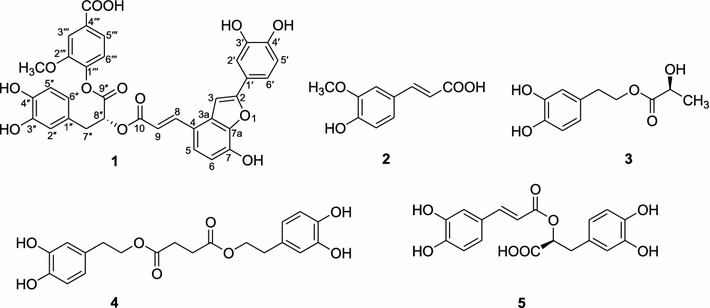
Chemical structures of compounds **1**–**5** isolated from *A. plantago*-*aquatica*

In continuation of our search for novel bioactive substances from this medicine plant, which has been proven to possess anti-chronic prostatitis activity, one new polyphenolic acid, plantain A (**1**), was isolated from *A. plantago*-*aquatica* by using various chromatographic methods, with four known phenolic compounds (**2**–**5**) (Fig. 1). The structures of the other isolated components ferulic acid (**2**), rynchopeterine A (**3**), rynchopeterine B (**4**) and rosmarinic acid (**5**) were determined by comparison to the ^1^H- and ^13^C-NMR spectral data in the literatures [10–12].

**Fig. 2:**
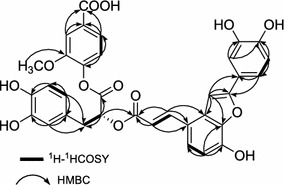
^1^H-^1^HCOSY and key HMBC correlations of **1**

**Fig. 3:**
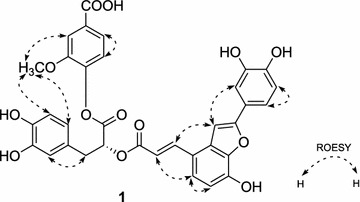
Key ROESY correlations of compound **1**

Compound **1**, which had the molecular formula C_34_H_26_O_13_, deduced from the positive-ion HR-ESIMS (*m*/*z* 665.1273 [M+Na]^+^) and ^13^C-NMR data. The ^1^H-NMR spectrum showed that the presence of a 3,4-dihydroxyphenyl lactic acid moiety [*δ*_H_ 6.70 (1H, d, *J* = 2.0 Hz, H-2″), 6.86 (1H, d, *J* = 8.0 Hz, H-5″), 6.60 (1H, dd, *J* = 8.0, 2.0 Hz, H-6″), 3.06 (1H, dd, *J* = 14.8, 4.0 Hz, H-7″a), 2.93 (1H, dd, *J* = 14.8, 8.8 Hz, H-7″b), 5.11 (1H, dd, *J* = 8.8, 4.0 Hz, H-8″)], a (*E*)-cinnamoyl moiety with three substituents in the benzene ring [*δ*_H_ 7.49 (1H, d, *J* = 8.4 Hz, H-5), 6.67 (1H, d, *J* = 8.4 Hz, H-6), 7.86 (1H, d, *J* = 16.0 Hz, H-8), 6.55 (1H, d, *J* = 16.0 Hz, H-9)], a three-substituted dihydrofuran [*δ*_H_ 6.73 (1H, s, H-3)], and a 3,4-dihydroxyphenyl [*δ*_H_ 7.41 (1H, d, *J* = 2.0 Hz, H-2′), 6.78 (1H, d, *J* = 8.4 Hz, H-5′), 7.38 (1H, dd, *J* = 8.4, 2.0 Hz, H-6′)], suggesting that **1** was a polyphenolic acid [13]. Additionally, the occurrence of a vanillic acid unit in the molecule could be easily deduced from the ^1^H- and ^13^C-NMR spectra [*δ*_H_ 7.52 (1H, d, *J* = 1.8 Hz), 7.48 (1H, d, *J* = 7.8 Hz), 7.36 (1H, dd, *J* = 7.8, 1.8 Hz), 10.78 (1H, s), and 3.75 (3H, s); *δ*_C_ 144.5, 151.3, 114.3, 129.2, 126.3, 123.0, 168.4, 55.9] [14]. Comparison of the ^1^H- and ^13^C-NMR data of **1** with those of salvianolic acid C (SAC) and vanillic acid displayed that the signals were substantially coincident [15]. All the above evidence combined with the detailed 2D-NMR analysis of ^1^H-^1^H COSY, HMBC and ROESY (Figs. 2, 3) correlations also implied that compound **1** was composed of SAC unit and vanillic acid unit. Moreover, the C-9″ carboxyl group of the SAC moiety was attached to the C-1′′′ hydroxy group of the vanillic acid. The structure of **1** is an ester dimer of SAC and vanillic acid between the hydroxyl group at C-1′′′ and the carboxylic acid group at C-9″. The suggestion was in accord with the observation of the chemical shift of C-9″ signal upfield shifted from *δ* 173.8 in SAC to *δ* 170.5 in **1** and the chemical shift of C-1′′′ signal upfield shifted from *δ* 151.2 in ADPP to 144.5 in **1** [16, 17]. This was further supported by ROESY correlations of 2′′′-OCH_3_ with H-5″ and H-6″ and acid hydrolysis of compound **1** with 10 N HCl gave SAC and vanillic acid, which was confirmed by HPLC analysis. Thus, the structure of **1**, which was established as shown in **1**, is a new phenolic compound, which we named plantain A.

### Biological assay

Experimental chronic non-bacterial prostatitis (CNP) was induced in rats by injecting carrageenan into prostate. Rats in drug-treated groups were administered the isolated compounds (**1**–**5**) or cernilton (positive control, i.e., reference standard) for 3 weeks while rats in normal and negative control groups were treated with saline at the same time. After treatment, the relative inflammatory factors, tumor necrosis factor-α (TNF-α), interleukin 1β (IL-1β), cyclooxygenase-2 (COX-2), prostaglandin E2 (PEG2), and transforming growth factor-β1 (TGF-β1) of the prostate tissues were measured by ELISA [2, 4].

As shown in Table 1, ELISA detection revealed that compounds **1** and **4** treatments obviously reduced TNF-α, IL-1β, PGE2, COX-2 and TGF-β1 levels compared with the control group. Compounds **1** and **4** markedly decreased the above inflammatory factors expression and showed significant anti-chronic prostatitis activity in rats.

**Table 1:** Effect of compounds **1**–**5** on TNF-α, IL-1β, PGE2, COX-2,TGF-β1 levels

Group	TNF-α (pg/mL)	IL-1β (pg/mL)	PGE2 (pg/mL)	COX-2 (pg/mL)	TGF-β1 (pg/mL)
Control	91.4 ± 6.1**	89.3 ± 7.2**	57.2 ± 9.3**	17.1 ± 3.7**	84.8 ± 9.9**
Negative control	173.8 ± 11.2	160.3 ± 10.1	130.2 ± 6.9	41.4 ± 1.9	133.1 ± 10.2
Cernilton	121.1 ± 10.5**	132.4 ± 9.7**	80.3 ± 5.7**	20.3 ± 2.4**	119.4 ± 11.7*
1	101.7 ± 9.9**	124.8 ± 8.0**	119.7 ± 10.9*	26.8 ± 4.1**	101.6 ± 9.7**
2	169.3 ± 11.7	156.7 ± 12.6	128.1 ± 11.7	39.7 ± 8.5	131.1 ± 12.2
3	161.1 ± 14.5	151.9 ± 10.3	125.7 ± 10.3	37.8 ± 6.2	129.8 ± 11.5
4	118.6 ± 10.3**	147.5 ± 11.2*	117.4 ± 8.3**	30.3 ± 1.8**	120.3 ± 11.4*
5	170.1 ± 9.4	159.9 ± 12.7	129.1 ± 11.9	40.1 ± 5.9	130.6 ± 10.3

Cernilton was tested at a dose of 30 mg/kg, the five compounds (**1**–**5**) were tested at a dose of 50 mg/kg

* p < 0.05, ** *p* < 0.01, significant as compared to the negative control group; Values are mean ± SD (n = 10)

## Experimental

### General procedure

NMR spectra were recorded on a Bruker AM-400 spectrometer (Bruker, Karlsruhe, Germany) using standard Bruker pulse programs. Chemical shifts are given as δ values with reference to tetramethylsilane (TMS) as internal standard. Column chromatography separations were carried out on silica gel (200–300 mesh, Qingdao Haiyang Chemical Co. Ltd, Qingdao, P.R. China), ODS (50 mesh, Merck China, Beijing, China), Diaion HP-20 (Pharmacia, Peapack, NJ, USA) and Sephadex LH-20 (Pharmacia, Peapack, NJ, USA). GF254 plates (Qingdao Marine, Qingdao, China) were used for thin layer chromatography, and spots were visualized under UV light or by spraying with 5% H_2_SO_4_ in ethanol followed by heating. All other chemicals used were of biochemical reagent grade.

### Plant material

Samples of *A. plantago*-*aquatica* were collected from Liuzhou City, Guangxi Province in China in May 2015. Taxonomic identification of the plant was performed by Professor Li-ping Xie. A voucher specimen (No. 20150701) has been deposited in the authors’ laboratory.

### Extraction and isolation

The dry *A. plantago*-*aquatica* (8 kg) were extracted two times under reflux with hot water (100 L × 3 h). After removing the solvent under reduced pressure, the residue was suspended in water and then sequentially extracted with petroleum ether, EtOAc and *n*-BuOH. The EtOAc extract (103 g) was subjected to silica gel column chromatography (CC) using CHCl_3_–MeOH (1:0–0:1) and divided into six fractions. Fraction 1 was separated by CC over silica gel using CHCl_3_–MeOH (9:1–7:3) and Sephadex LH-20 CC using MeOH to obtain **2** (24 mg) and **3** (27 mg). Fraction 3 was separated by CC on Si gel using CHCl_3_–MeOH (8:2–6:4) to give subfraction 3–1 (5.5 g), subfraction 3–2 (6 g) and subfraction 3–3 (12 g). Subfraction 3–3 was purified by semi-preparative HPLC to afford compounds **1** (20 mg), **4** (27 mg), and **5** (30 mg).

### Characterization of plantain A (1)

Obtained as brown amorphous powder, [*α*]_D_^25^ + 66.9° (*c* 0.10, MeOH); HR-ESIMS *m*/*z* 665.1273 (C_34_H_26_O_13_Na [M+Na]^+^, Cal. 665.1271); IR *v*_max_ (KBr): 3433, 2940, 1601, 1524, 1446, 1360, 1282, 1192, 1110, and 1066 cm^−1^. ^1^H-NMR and ^13^C-NMR (DMSO-*d*_*6*_) data see Table 2 (For further information, see Additional file 1).

**Table 2:** ^13^C- and ^1^H-NMR data of **1** in DMSO-*d*_*6*_ (400 MHz for H, 100 MHz for C)

No.	C	H	No.	C	H
1			1″	131.5	
2	157.8		2″	117.8	6.70 (1H, d, *J* = 2.0 Hz)
3	99.2	6.73 (1H, s)	3″	146.1	
3a	127.9		4″	145.4	
4	120.6		5″	115.5	6.86 (1H, d, *J* = 8.0 Hz)
5	133.9	7.49 (1H, d, *J* = 8.4 Hz)	6″	121.4	6.60 (1H, dd, *J* = 8.0, 2.0 Hz)
6	111.4	6.67 (1H, d, *J* = 8.4 Hz)	7″	36.6	3.06 (1H, dd, *J* = 14.8, 4.0 Hz)
7	147.4				2.93 (1H, dd, *J* = 14.8, 8.8 Hz)
7a	142.8		8″	73.5	5.11 (1H, dd, *J* = 8.8, 4.0 Hz)
8	145.7	7.86 (1H, d, *J* = 16.0 Hz)	9″	170.5	
9	119.5	6.55 (1H, d, *J* = 16.0 Hz)	1′′′	144.5	
10	166.7		2′′′	151.3	
1′	120.6		3′′′	114.3	7.52 (1H, d, *J* = 1.8 Hz)
2′	112.9	7.41 (1H, d, *J* = 2.0 Hz)	4′′′	129.2	
3′	147.8		5′′′	126.3	7.48 (1H, d, *J* = 7.8 Hz)
4′	148.8		6′′′	123.0	7.36 (1H, dd, *J* = 7.8, 1.8 Hz)
5′	116.5	6.78 (1H, d, *J* = 8.4 Hz)	COOH	168.4	10.78 (1H, s)
6′	117.2	7.38 (1H, dd, *J* = 8.4, 2.0 Hz)	OCH_3_	55.9	3.75 (3H, s)

### Acid hydrolysis of plantain A (1)

A solution (3 mg) of **1** in 10 N HCl (1.5 mL) was heated at 100 °C for 5 min under an N_2_ atmosphere. After cooling, the solution was removed. The residue was dissolved with methanol, stirred at 45 °C for 10 min. The methanol solution was analyzed by HPLC using Hypersil C_18_ (250 mm × 4.6 mm). The HPLC linear gradient profile was as follows: water (containing 0.5% phosphoric acid), acetonitrile (containing 0.5% phosphoric acid) 54:46 v/v (0–15 min), 54:46–20:80 (15–20 min), and 20:80 (20–30 min) at a flow-rate of 1 mL/min. The separation was carried out at 25 °C. Compounds were analyzed 286 nm. The peak identity of each component was confirmed by comparison of the retention time. Retention times of SAC, plantain A, and vanillic acid were 17.15, 20.52 and 10.08 min.

### Animals

Eight weeks old male Wistar rats (220–250 g) were provided by the Laboratory Animal Center of Zhejiang University (Certificate no. SYXK 2012-0178). The animals had free access to feed and water, and were allowed to acclimatize for at least 1 week before use. The drugs were dissolved in water, and administered using a 5 mL syringe with a 4 cm long gavage needle through the mouth once daily for 3 weeks.

### Biochemical assays

Chronic non-bacterial prostatitis were induced as previously described. Prostates of rats in control group were injected with 0.1 mL saline by an injector, and the same volume of 1% carrageenan in rats of other groups. Seven days after preparing the model rats of chronic nonbacterial prostatitis, rats in sample group, they were orally administered compounds **1**–**5**, while rats in positive (reference standard) group were. Administered cernilton, both groups for 3 weeks. Rats of normal and negative control groups were administered saline at the same time [2, 4].

After the rats were sacrificed by cervical dislocation, the pro-inflammatory cytokines TNF-α and IL-1β of prostate tissues of all rats were measured by commercial ELISA assay kits, according to manufacturer’s instruction. The samples and standards were all run in duplicates and the data were then averaged. The results were expressed as pg/mL.

PGE2, COX-2, and TGF-β1 were measured in prostate tissues using commercial ELISA kits. All assays were performed in 10% prostate supernatant in accordance with manufacturer’s instructions. The levels of PGE2, COX-2, and TGF-β1 in prostate tissue are expressed in pg/mL [1, 2].

### Statistical analysis

Data analysis was performed by one-way analysis of variance with the Dunnett’s post hoc test for multiple comparisons by SPSS 10.0 software. Data were expressed as the mean ± standard error of the mean (SEM). The level of statistical significance was set at *p* < 0.05 (Additional file 1).

## Additional file

**Additional file 1.** **Figure S1** ^1^H NMR spectrum (400 MHz, DMSO-*d*6) of compound **1**. **Figure S2** ^13^C NMR spectrum (400 MHz, DMSO-*d*6) of compound **1**. **Figure S3** ^1^H-^1^H COSY spectrum (400 MHz, DMSO-*d*6) of compound **1**. **Figure S4** HMBC spectrum (400 MHz, DMSO-*d*6) of compound **1**. **Figure S5** HR-ESIMS spectrum of compound **1**.
