# Supplementary material for: Phenolic constituents from Alisma plantago-aquatica Linnaeus and their anti-chronic prostatitis activity
Source: Chem Cent J. 2017 Nov 21;11:120. doi: 10.1186/s13065-017-0350-9 (PMC5696274; doi:10.1186/s13065-017-0350-9)
Supplement: Supplementary file 1 — Additional file 1. Figure S1 1H NMR spectrum (400 MHz, DMSO-d6) of compound 1. Figure S2 13C NMR spectrum (400 MHz, DMSO-d6) of compound 1. Figure S3 1H-1H COSY spectrum (400 MHz, DMSO-d6) of compound 1. Figure S4 HMBC spectrum (400 MHz, DMSO-d6) of compound 1. Figure S5 HR-ESIMS spectrum of compound 1. [file 13065_2017_350_MOESM1_ESM.doc]

**Supplementary Materials**

**Phenolic constituents from *Alisma plantago-aquatica* Linn. and their anti-chronic prostatitis activity**

Ya-sheng Huang^1,2^, Qi-qi Yu^2^, Yin Chen^2^, Min-jie Cheng^2^, Li-ping Xie^1*^

The original NMR and HR-ESIMS spectra of compound **1** are listed as **Figures S1-S5:**


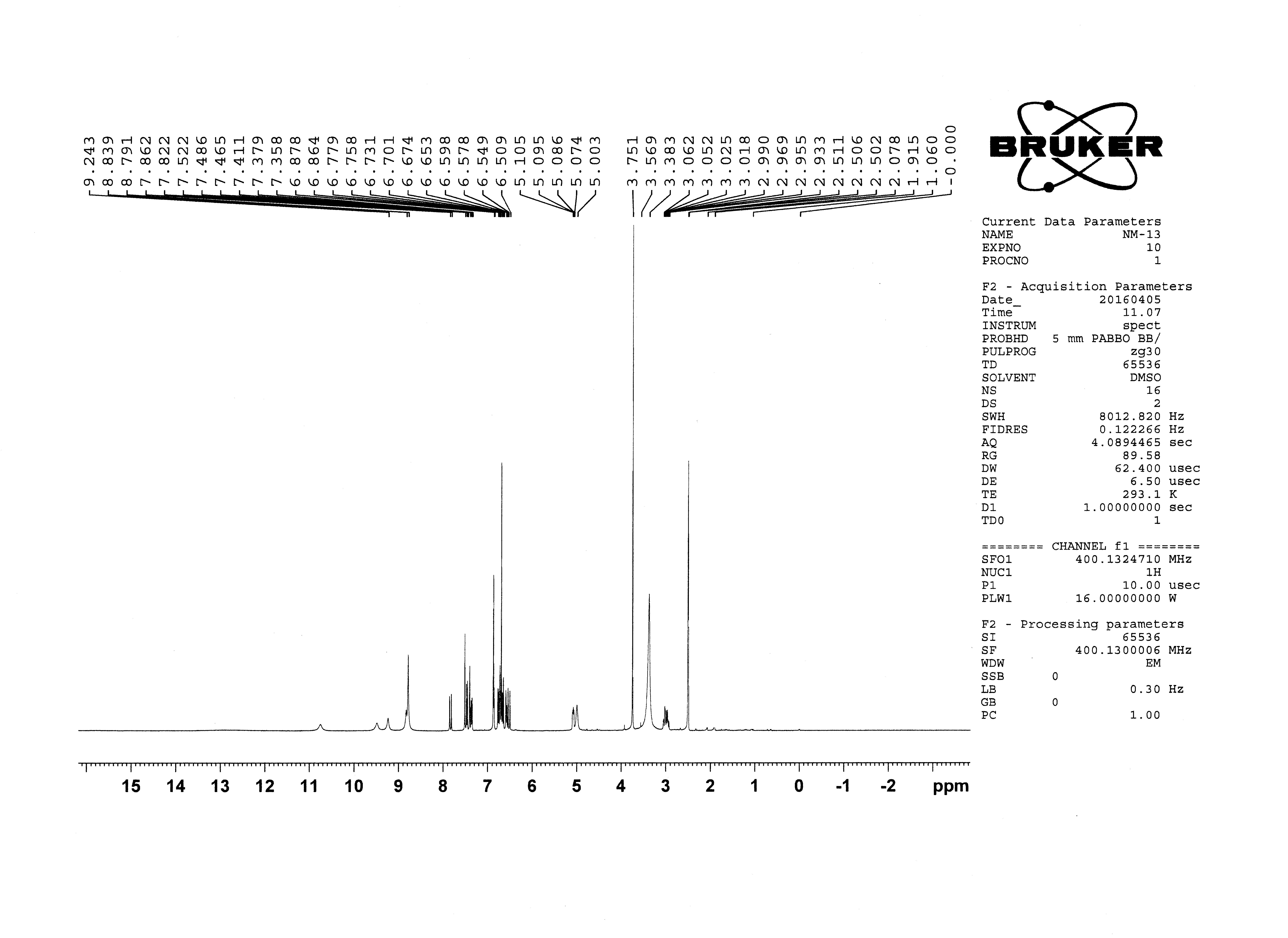


**Figure S1** ^1^H NMR spectrum (400 MHz, DMSO-*d*6) of compound **1**


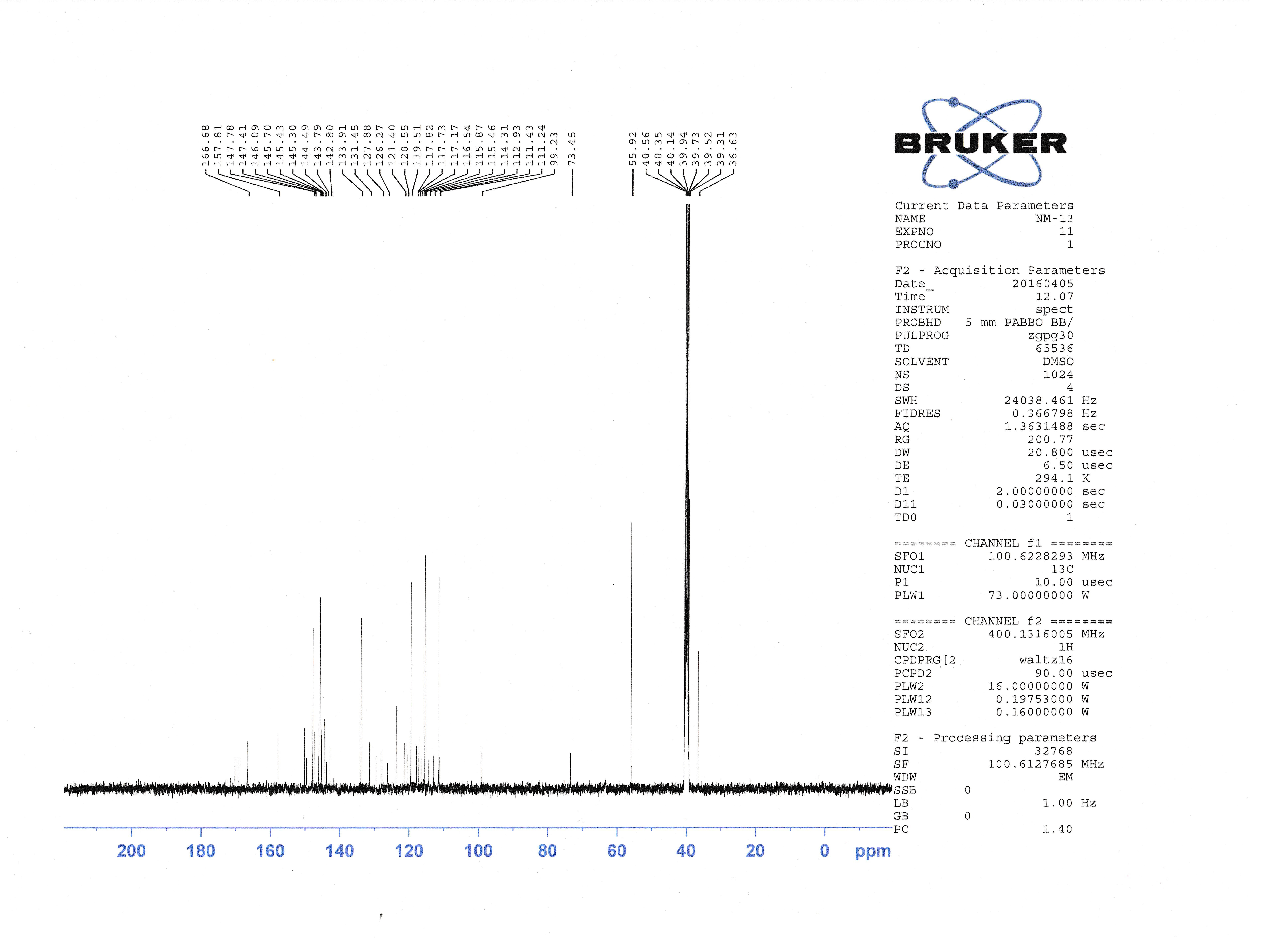


**Figure S2** ^13^C NMR spectrum (400 MHz, DMSO-*d*6) of compound **1**


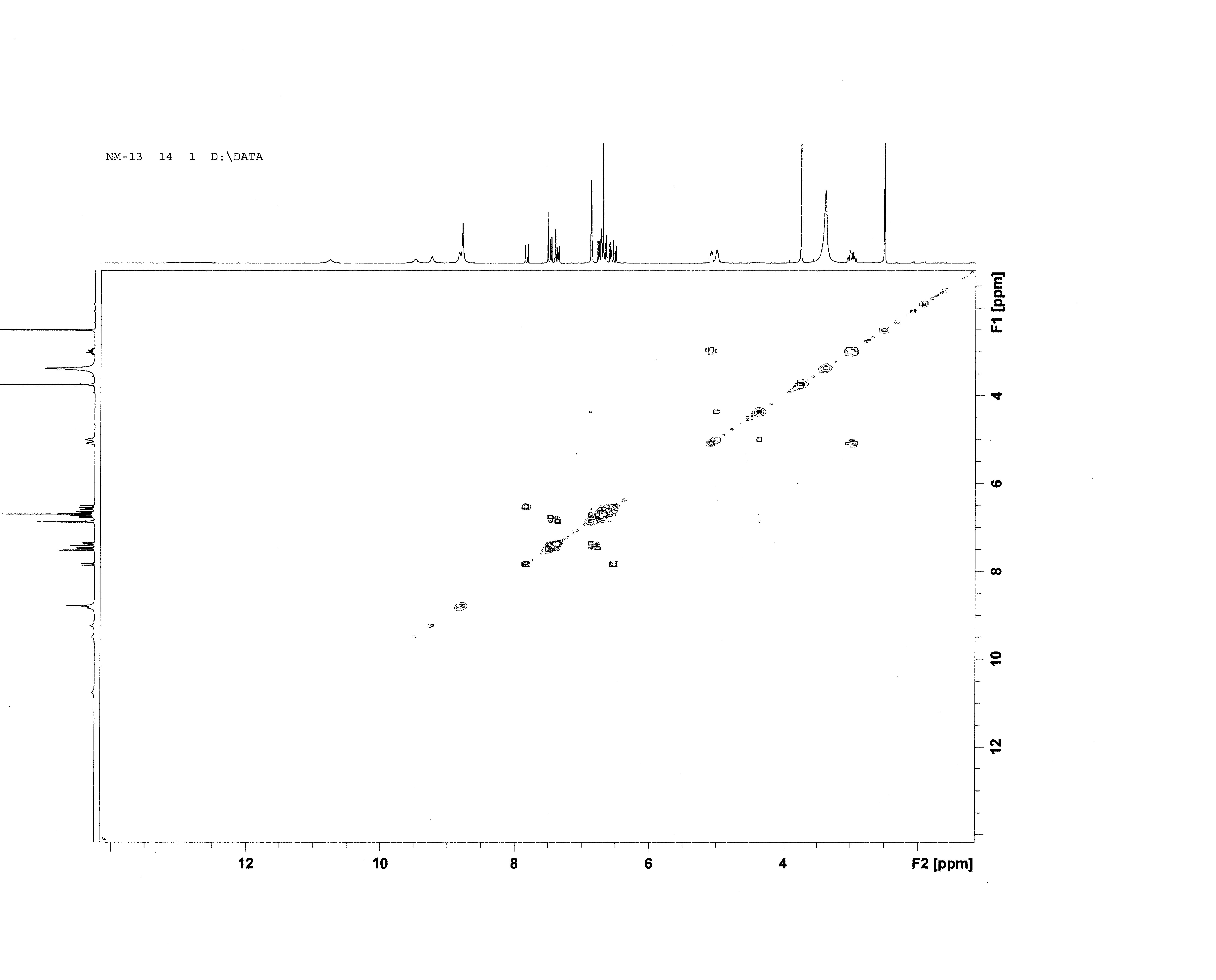


**Figure S3** ^1^H-^1^H COSY spectrum (400 MHz, DMSO-*d*6) of compound **1**


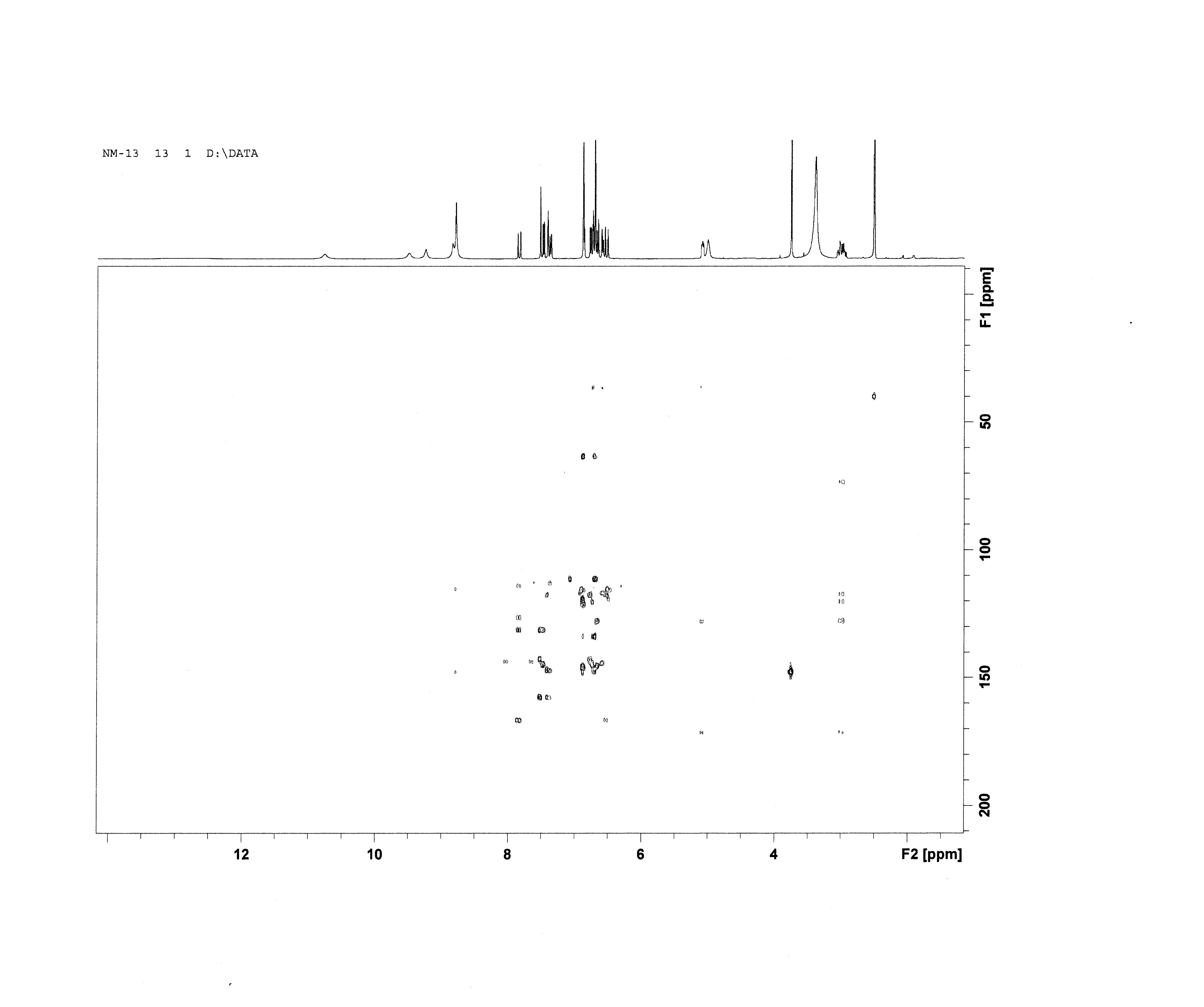


**Figure S4** HMBC spectrum (400 MHz, DMSO-*d*6) of compound **1**


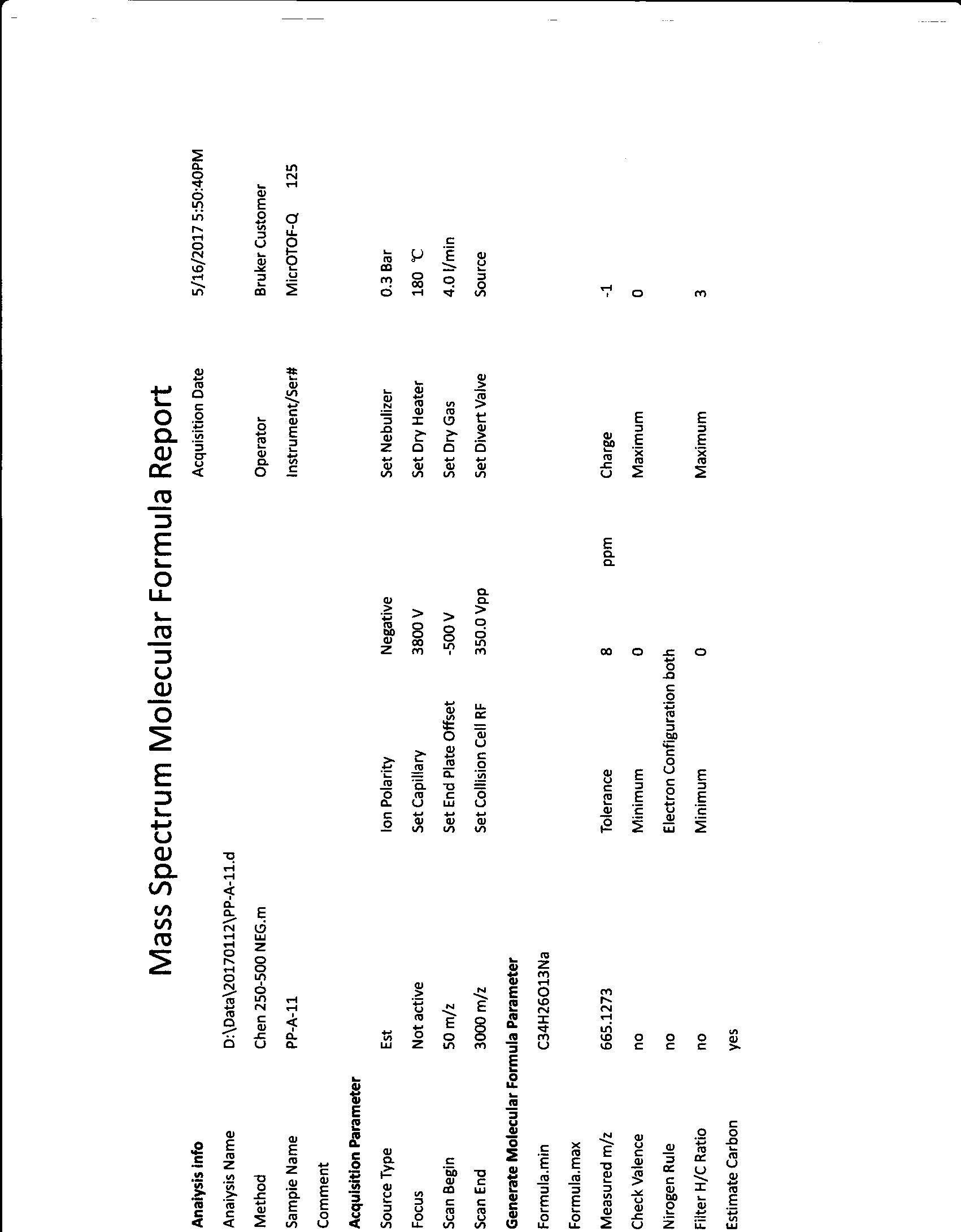


**Figure S5** HR-ESIMS spectrum of compound **1**
